# Osteolytic mystery: A rare case of pathologic fracture from a phosphaturic mesenchymal tumor in hip and femur

**DOI:** 10.1016/j.radcr.2024.07.036

**Published:** 2024-07-30

**Authors:** Murad Aldoghmi, Erwin Ho, Ryan O'Connell, Roozbeh Houshyar

**Affiliations:** Department of Radiological Sciences, University of California Irvine, Building 1, 101 The City Dr S, Orange, CA 92868 USA

**Keywords:** Phosphaturic mesenchymal tumor, Osteomalacia, Multiple myeloma, Neoplasm, Connective tissue, Hypophosphatemia

## Abstract

Phosphaturic mesenchymal tumor (PMT) is a rare tumor causing bone complications and myopathy. Histologically, PMT displays a mix of spindled cells, osteoclast-like giant cells, basophilic matrix, and flocculent or “grungy” calcification. Here we describe a case of PMT in the right hip and proximal femur, initially suspected to be multiple myeloma, presenting with osteolytic lesions and elevated alkaline phosphatase. Tests for malignancy were negative, but a subsequent biopsy confirmed PMT. The patient underwent hip biopsy, femur resection, and hemiarthroplasty, with follow-up MRI recommended.

## Introduction

Tumor-induced hypophosphatemic osteomalacia (TIO) is a rare syndrome caused by tumors producing phosphatonins, resulting in bone pain, fractures, and myopathy [1]. This is due to an overproduction of fibroblast growth factor 23 (FGF23) from benign mesenchymal tumors, leading to disruptions in renal metabolism, lowered vitamin D3 concentration, and decreased phosphate reabsorption [[2], [3], [4]]. Elevated serum FGF23 levels can diagnose TIO, with octreotide sestamibi scans locating the tumor [5]. TIO is often missed initially due to nonspecific symptoms and its similar clinical picture to multiple myeloma and other osteopathic disorders [4].

Weidner and Cruz's 1987 study introduced “phosphaturic mesenchymal tumor, mixed connective tissue variant” (PMT) to describe tumors with specific histological features. PMT is rare, fewer than 500 cases have been reported in the literature [6]. World Health Organization (WHO) recognized PMT in 2013 and found them related to FN1-FGFR1/FGF1 genes. PMT involves the extremities, acral sites, appendicular skeleton, cranial bones, and paranasal sinuses [7]. While they cause chronic hypophosphatemia, most PMTs are benign. However, malignant versions can lead to metastasis and poor outcomes [8]. Histologically, PMT displays a mix of spindled cells, osteoclast-like giant cells, basophilic matrix, and flocculent or “grungy” calcification. Malignant PMT cases have shown more aggressive profiles, but most are benign. In patients with PMT, the female to male ratio stands at 1:1.2, with the mixed connective tissue subtype representing over 70% of cases [9].

## Case presentation

**Fig. 1:**
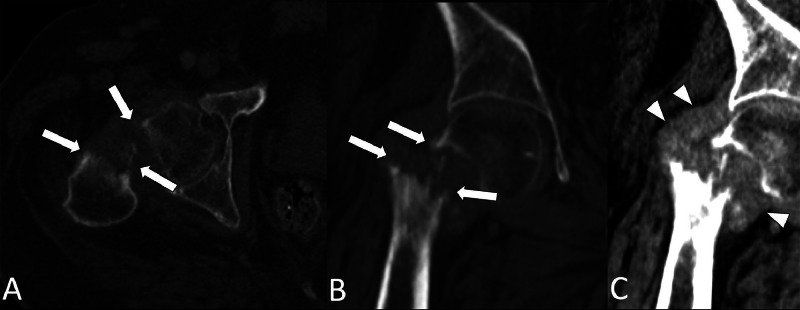
Axial and coronal CT images (A-B) of the pelvis shows a pathologic fracture of the right hip (white arrows). Coronal view (C) shows a surrounding soft tissue mass (arrowheads).

The patient is a 49-year-old male with a history of severe, bilateral knee osteoarthritis. At baseline, he takes Oxycodone, Paracetamol for pain and ambulates with a walker. One week before his initial visit, he developed increasingly severe pain in his right hip that impaired his ability to walk. Outside computed tomography (CT) scan of the chest, abdomen, and pelvis showed right femoral transcervical neck fracture with a large surrounding soft tissue mass and multiple lytic lesions of the axial and appendicular skeleton (Fig. 1). In the absence of trauma or an inciting event, the CT findings were highly concerning for a pathologic fracture secondary to multiple myeloma or metastatic processes. He was subsequently transferred to our institution for further evaluation by orthopedic oncology.

Initial physical examination revealed pain of the right hip with log roll, intact sensation to light touch bilaterally, and unpalpable distal pulses. Laboratory results were significant for elevated alkaline phosphatase (ALP) of 259 U/L but notably negative for anemia, reduced glomerular filtration rate, or calcium imbalance. Further, diagnostic work-up for multiple myeloma including serum protein electrophoresis, urine protein electrophoresis, quantitative immunoglobulins were within normal limits except for mildly elevated serum kappa free light chains at 24.1 mg/L. Malignancy biomarkers such as CA 19-9, PSA, CEA, TSH, and PTH were unremarkable. Underlying lytic lesion with soft tissue component centered around the femoral head and neck region was seen on X-Ray hip imaging, with additional osteolytic lesions of the innominate bones, sacrum, lower lumbar spine, and bilateral femora.

Negative workup for multiple myeloma and malignancy biomarkers lead to the consideration of other differentials including sarcoidosis, autoimmune, or infectious. Infectious work up was negative for Coccidioides, syphilis, tuberculosis, Brucella, Histoplasma and Bartonella henselae.

**Fig. 2:**
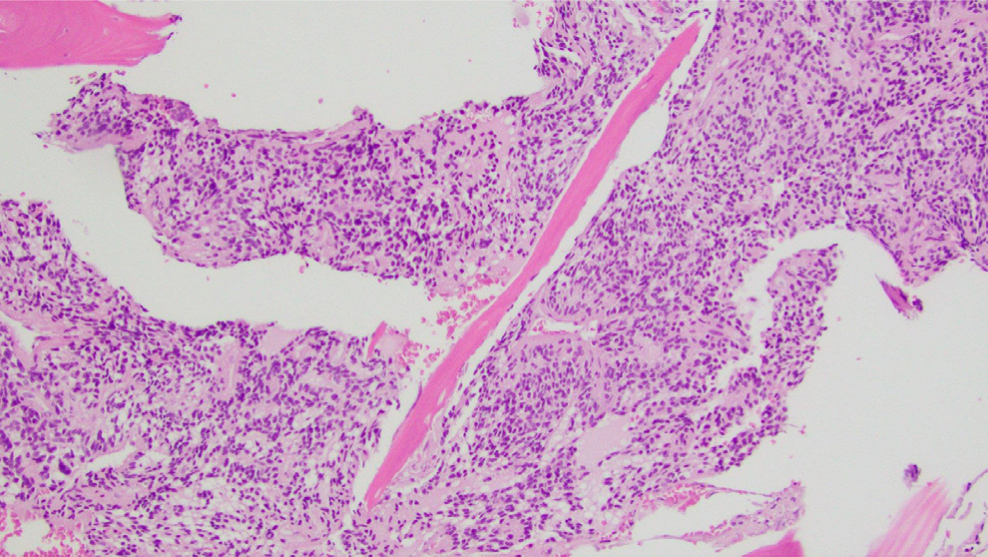
A 100x magnification H&E stained tissue: This image highlights the invasive appearance of the tumor, encasing trabecular bone.

**Fig. 3:**
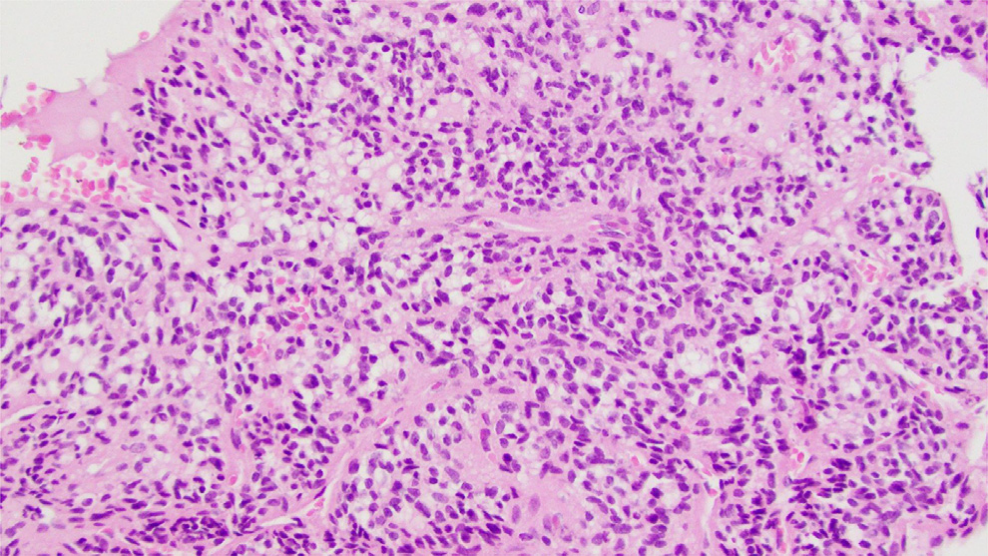
A 200x magnification H&E stained tissue: This image illustrates the spindled to ovoid histologic appearance, mild to moderate nuclear pleomorphism, and low mitotic rate.

**Fig. 4:**
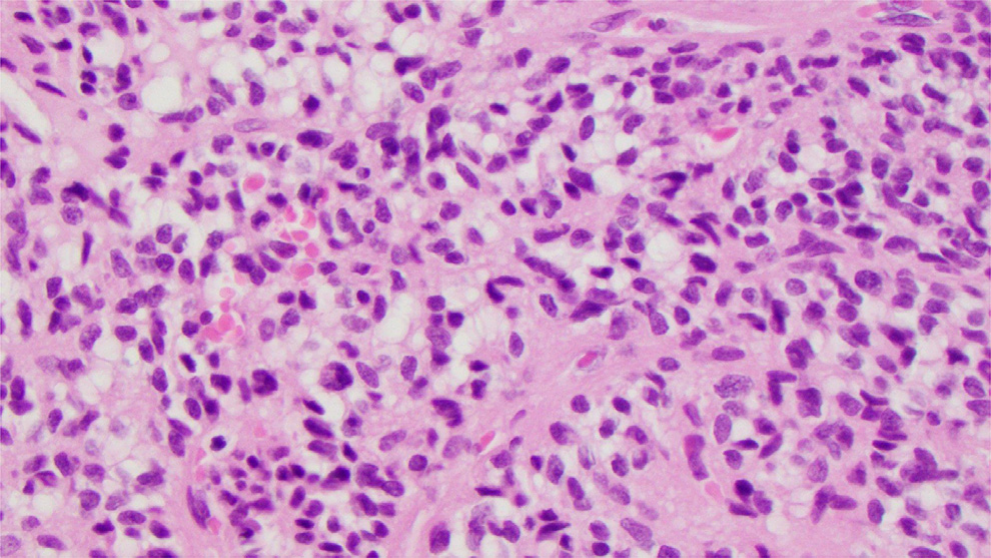
A 400x magnification H&E stained tissue: This image illustrates the spindled to ovoid histologic appearance, mild to moderate nuclear pleomorphism, and low mitotic rate.

**Fig. 5:**
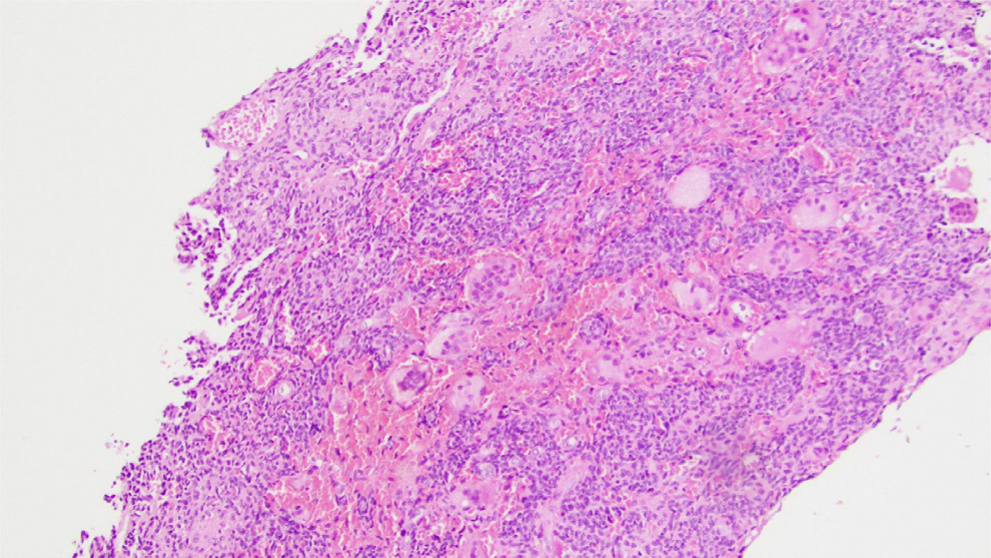
A 100x magnification H&E stained tissue: This image shows the abundant multinucleated giant cells within the lesion.

Right hip open biopsy with proximal femur resection and hemiarthroplasty planned by Ortho and internal medicine. Preliminary biopsy results revealed a small blue cell tumor encasing trabecular bone with oval to spindled morphology within the right proximal femur (Fig. 2, Fig. 3, Fig. 4). Abundant multinucleated giant cells were also seen within the lesion (Fig. 5). Immunohistochemistry was positive for ERG and SATB2. No grungy calcification was identified. Differential diagnoses include but is not limited to osteosarcoma, high-grade chondrosarcoma, and phosphaturic mesenchymal tumor. Orthopedic oncology recommended follow up PET CT scan which revealed only nonspecific postsurgical changes with activity along the medial aspect of the femoral stem.

In-situ hybridization revealed increased FGFR1 and FGF23. Both markers lead to decreased renal phosphate reabsorption and 1,25-dihydroxyvitamin D production. High levels are reported in mesenchymal tumors. The combination of these findings is indicative of phosphaturic mesenchymal tumor, confirming diagnosis. Given the low mitotic rate and low Ki-67 of the patient's femoral tumor, he is not suited for the systemic chemotherapy typically reserved for higher-grade tumors. Patient was discharged when stable with recommendations to follow-up with outpatient MRI.

## Discussion

This case of PMT is unique due to the presence of multiple osteolytic lesions, generally suggestive of multiple myeloma, resulting in pathologic fracture accompanied by lab findings including elevated alkaline phosphatase (ALP) and mildly increased serum kappa free light chains, but lacking signs of anemia, diminished glomerular filtration rate, or calcium imbalances. Next-generation sequencing (NGS) showed an appreciable rise in FGFR1 and FGF23, affirming the diagnosis of a phosphaturic mesenchymal tumor. The lesion is hypercellular, enveloping the inherent trabecular bone. These cells exhibit mild to moderate nuclear variability, with a low mitotic rate, devoid of necrosis, and containing abundant multinucleated giant cells. While the predominant stroma is fibrous or hyalinized, sections display chondroid formation without the typical “grungy” calcification, and the tumor cells are observed encircling blood vessels. A comprehensive immunohistochemical examination revealed negative results for many stains (CAM5.2, CK7, CK20, EMA, AE1/AE3, CD45, CD3, CD20, C138, MUM1, S100, SOX10, HMB45, Melan-A, STAT6, TLE-1, SATB2, CD99, CD31, and CD34), with only SATB2 and ERG displaying positivity in lesion cells. No mutations were detected in the IDH1/IDH2 analysis. In this case, the soft tissue mass mesenchymal tumor likely led to a pathologic fracture of the right hip with subsequent sequelae of symptoms (hip pain, difficulty ambulating).

TIO is a rare condition, with about 1000 cases reported globally, and due to its nonspecific symptoms, over 95% of cases are often misdiagnosed [3,10,11]. Its primary pathological mechanism stems from the tumor's unchecked production of FGF23, leading to chronic hypophosphatemia and reduced bone mineralization [4,12,13]. A retrospective study revealed that the most prevalent symptoms include bone pain, walking difficulties, fractures, and muscle weakness [10]. Many TIO patients, like those from PMT, traverse various specialties before accurate diagnosis, emphasizing the importance of a comprehensive medical history, physical exams, and laboratory tests [4,11,14]. Serum tests typically show abnormally low phosphorus levels consistent with FGF23’s effects [14,15]. Imaging, such as 68Ga-DOTA-based PET/CT scans, is crucial for detection, and complete surgical resection of PMTs often results in recovery [14,16,17]. Notably this rare pathology, especially seen in this case, necessitates clinician awareness for prompt recognition and treatment consistent monitoring to preempt complications.

## Conclusion

PMT is a rare underlying cause of pathologic fractures and metabolic dysfunction. It can present with nonspecific symptoms and osteolytic lesions, thus resembling numerous established etiologies, such as multiple myeloma. When initial laboratory findings do not support suspected causes of pathologic fracture, PMT should be considered amongst potential differential diagnoses. This case report aims to advance awareness and understanding of PMT for accurate diagnosis and improved patient care.

## Patient consent

A written and informed consent was obtained from the patient for publication of this case report.
